# Variable selection for causal inference, prediction, and descriptive research: a narrative review of recommendations

**DOI:** 10.1093/ehjopen/oeaf070

**Published:** 2025-06-04

**Authors:** Brett P Dyer

**Affiliations:** Griffith Health, Griffith University, Gold Coast, 4222 QLD, Australia

**Keywords:** Variable selection, Causal inference, Prediction, Prognostic factor, Descriptive epidemiology, Methods

## Abstract

There is a growing appreciation that the methods and analyses of medical studies should be tailored towards the type of research question. However, frequent conflation exists with respect to the reasons for statistically adjusting for variables in analyses and the methods that should be used for variable selection in regression models. Non-randomized causal studies require statistical adjustment for confounders that may bias the causal effect estimate. Predictor/prognostic factor studies may present unadjusted associations and/or present associations statistically adjusted for existing predictors to establish the added predictive value of the candidate predictor over and above known predictors. Prediction models aim to identify a set of variables that are clinically useable and are collectively the best at predicting the outcome. Descriptive studies may want to characterize the outcome distribution with respect to an additional variable or standardize with respect to a nuisance variable for which the study sample differs from the target population. This narrative review summarizes background theory and existing advice on how variable selection should differ for causal research, prediction modelling, predictor/prognostic factor research, and descriptive research. Examples of variable selection approaches from published cardiovascular research are also provided.

## Introduction

In observational research, statistical adjustment for covariates is often integral to the validity of a study. However, variable selection should not be a ‘one-size-fits-all’ procedure. In prediction research, causal/explanatory research, and descriptive research, the quality of statistical analyses in published manuscripts has been questioned.^1–3^ A key factor driving poor data analysis is the frequent conflation between different types of medical research.^4^ In particular, the differences between prediction research and causal research have been highlighted,^5–11^ and more recently, researchers have called attention to the conflation between descriptive and causal research.^2,11,12^ These existing articles focus on the differences in study aim, research questions, interpretation of results, and the implications of conflating aetiology, prediction, and description. Model selection is one key area of difference between prediction, causal inference, and descriptive research and is one of the main reasons for conflation.^4^ Most modern epidemiological textbooks focus on either confounder adjustment^13–15^ or prediction model development^16,17^ when teaching variable selection. In the past 5 years, articles in high-impact journals have encouraged a more united framework for teaching description, prediction, and causal inference, so researchers can appreciate the theoretical and methodological differences between the three types of research.^5,18–20^ This narrative review summarizes existing advice from the literature to highlight how variable selection should differ according to the type of medical research, i.e. in causal inference, prediction modelling, predictor/prognostic factor research, and descriptive research.

### The different classes of medical research

It has been suggested that medical research can be categorized into three classes: description, prediction, and causal inference.^5,6,10,11^ Descriptive research aims to quantitatively describe the distribution of disease (or other features of health) in a population. For example, the incidence, prevalence, or average time to event for a defined population (or populations) over a specified period of time may be estimated to describe the occurrence of disease, to whom it occurs, over what time it occurred, and where it occurred.^21^ Descriptive research can help to analyse trends, generate hypotheses for analytical studies, or guide policymakers in planning health services and deciding how to utilize resources. Most literature refers to prediction research as one class of medical research; however, it can be broken down into two smaller subclasses, which I refer to as predictor research and prediction modelling, each of which requires a different variable selection approach. The two general aims of prediction research are either to understand whether a single factor predicts an outcome (i.e. predictor research) or to use a combination of predictors to estimate an individual’s risk of the outcome (i.e. prediction modelling). Health outcomes of interest in prediction studies could be the presence of disease, the occurrence of a disease, or the future health status of someone with a disease (i.e. the patient’s prognosis).^22^ This information about the patient’s likely health status can be used to inform clinical decision-making. The aim of causal research can be to understand the causal mechanism that leads to a health-related outcome and/or to estimate the causal effect of an exposure or intervention on an outcome.^23^ Causal research may provide insight into aetiology, identify a target for intervention, or assess the effectiveness of an intervention.

### Causal modelling

In causal modelling, the purpose of adjusting for confounders is to obtain exposed and unexposed groups that, within strata of the set of confounders, would have the same average risk of the outcome for each level of the exposure.^24^ No unmeasured confounding is one necessary assumption required to estimate the average causal effect of an exposure on an outcome. No unmeasured confounding is one component of the exchangeability assumption—one of three ‘identifiability assumptions’ from the potential outcomes framework.^14^ Methodological developments in previous decades have helped to clarify how covariate adjustment sets should be selected and the conditions that must be met to sufficiently control for confounding. This section focuses on using causal directed acyclic graphs (DAGs) with the backdoor criterion for attempting to estimate causal effects, since they are a common approach in observational studies; however, alternative approaches may also be used, e.g. instrumental variable analysis^25^ and the front-door criterion.^26^

Due to data-driven methods being agnostic to temporal ordering and the causal structure of the data-generating process, data-driven methods are not appropriate for causal modelling; instead, subject matter knowledge must be used. DAGs^27,28^ are the most commonly used approach for summarizing background knowledge and assumptions about the causal relationships among an exposure, outcome, and covariates. Implied statistical independencies can also be read off a DAG and can guide researchers about which variables should, and should not, be conditioned on in order to estimate a causal effect. To aid the decision-making about covariate adjustment, researchers can use a DAG to determine the implications that conditioning on different types of covariates will have on the estimation of the causal effect. Below is an overview of the essentials of DAGs and covariate adjustment using the backdoor criterion. More comprehensive guides on the use of DAGs are available in Glymour *et al*.,^29^ Digitale *et al*.,^30^ and Tennant *et al*.^31,32^

**Figure 1:**
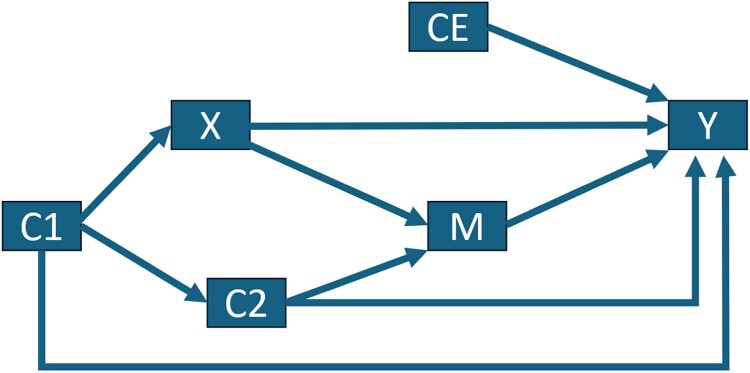
A directed acyclic graph to illustrate covariate roles.

Unidirectional arrows are used in causal DAGs to depict that the variable at the tail of the arrow *could potentially* directly have some non-zero causal effect on the variable at the arrowhead.^31^ In addition to direct causes, DAGs can show indirect effects through causal paths. A causal path is a chain of arrows all flowing in the same direction, e.g. X→M→Y in *Figure 1*.^29,30^ A path in which not all arrows are flowing in the same direction is called a non-causal path, e.g. X→M←C2→Y and X←C1→Y in *Figure 1*.^29,30^ Paths can be either blocked or open. A blocked path between two variables will not transmit a statistical association between those variables, whereas an open path can transmit a statistical association between the variables.^27,31^ Conditioning on a variable on a causal path will block that path, whereas conditioning on a variable on a non-causal path can have different implications according to whether the non-causal path contains a collider.^27^ A collider is a variable where two arrowheads collide/meet, e.g. X→M←C2 in *Figure 1*. Conditioning on a variable on a path that does not contain a collider, e.g. X←C1→Y, will block it.^27^ Conditioning on a collider, or a variable caused by the collider, will unblock the non-causal path, which may induce collider bias, i.e. the introduction of a non-causal association that may bias a causal effect estimate.^33,34^ For example, conditioning on M in *Figure 1* could unblock the non-causal path X→M←C2→Y and transmit a non-causal association between X and Y.

A backdoor path from an exposure X to an outcome Y can be defined as a path from X to Y that has an arrow pointing into X, e.g. X←C1→Y or X←C1→C2→Y.^27^ Backdoor paths are non-causal paths, and all backdoor paths need to be blocked to avoid confounding (a bias that can arise due to common causes of an exposure and outcome.^14^) Conditioning on a variable along the backdoor path (through statistical adjustment in a regression model, or otherwise) can block the non-causal association from being transmitted (unless that path is already blocked by a collider). Thus, adjustment for C1 would block the path X←C1→Y, and adjustment for C1 and/or C2 would block X←C1→C2→Y.

Mediators lie on the causal path between the exposure and outcome (e.g. X→M→Y), so they should not be conditioned on since this would prevent the causal association between the exposure and outcome from being transmitted. If a mediator is a collider, then it may induce collider bias if conditioned on, as previously demonstrated with M in *Figure 1*. Competing exposures (variables which cause the outcome but are not associated with the exposure) do not transmit an association between the exposure and outcome, so they do not affect bias but can affect the precision of the effect estimate.^35^

As described above, there are various types of structures in a DAG that can be identified to determine the implications that conditioning on different covariates can have on bias when estimating a causal effect. The structures described in this article are important for understanding the backdoor criterion^27,34^—a criterion which can be applied to a DAG to determine whether a statistical adjustment set is sufficient to condition on in order to estimate a causal effect. The criterion states that, in order to estimate the causal effect of an exposure X on an outcome Y, the statistical adjustment set should block all paths between X and Y that contain an arrow into X in the DAG (i.e. backdoor paths), and statistical adjustment should not be made for variables directly or indirectly caused by X.^27,34^ In terms of *Figure 1*, the unblocked paths from X to Y that contain an arrow into X are X←C1→Y, X←C1→C2→M→Y, and X←C1→C2→Y. Statistical adjustment for C1 alone, or C1 and C2, would be sufficient to block these paths. M is caused by X, so it should not be adjusted for. The online software DAGitty can be used to identify causal paths, biasing paths, and implied statistical independencies from user-drawn DAGs, as well as testing whether proposed statistical adjustment sets are sufficient.^36,37^

A limitation of the backdoor criterion is that it relies on the correct specification of the DAG; thus, any author uncertainty about DAG construction must be reported clearly. It has been highlighted that the way DAGs are constructed and reported in applied health research is inconsistent.^31^ Thus, researchers should follow recommendations (such as those provided by Tennant *et al*.^31^) to ensure that DAGs are constructed in a methodologically rigorous way that is consistent across studies.

When working with observational data, there is always a concern of residual confounding, and researchers may question whether there is an unknown confounder that they have missed from their DAG. To assess the impact that unmeasured confounders may have on the effect estimate and the study’s conclusions, sensitivity analyses for unmeasured confounding should be conducted to understand how strong the potential unmeasured confounding must be to explain away the effect estimate (*Box 1*).^38–40^

**Figure 2:**
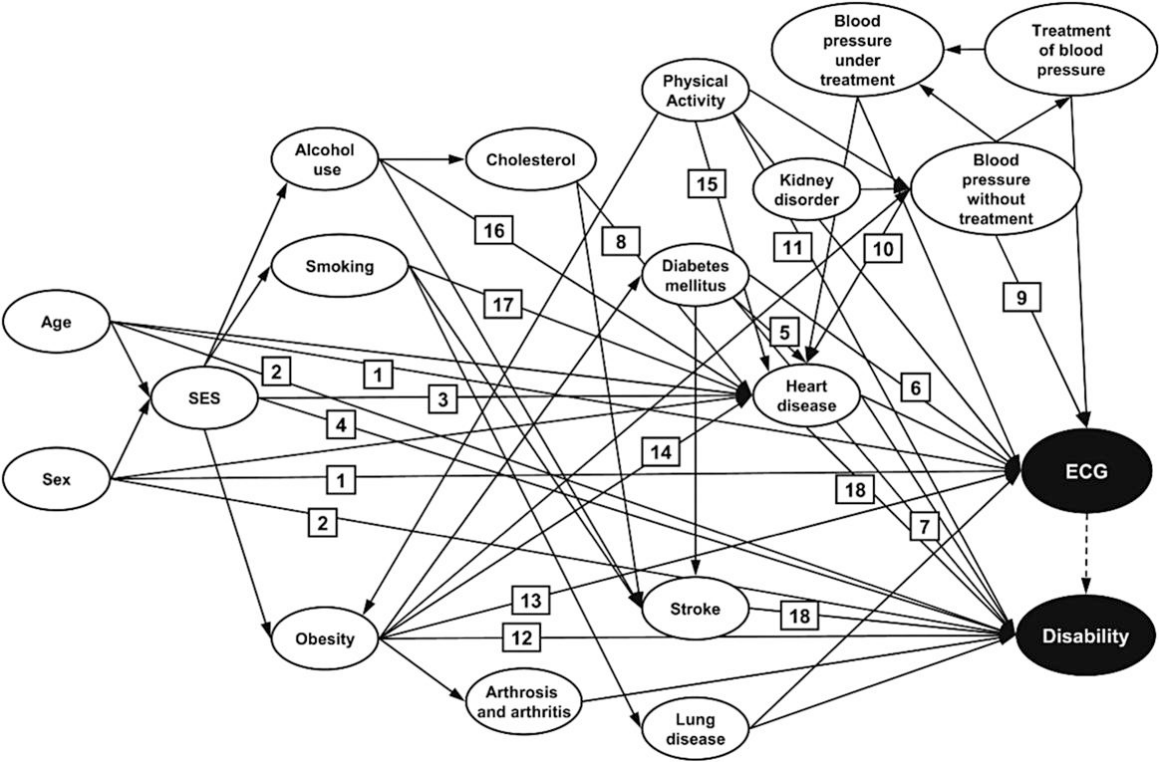
A directed acyclic graph to identify confounders of the association between electrocardiographic findings and disability. Numbered references to provide empirical evidence of some effects are included from the article by Röhrig *et al*. The authors only included arcs for major associations with age and sex to avoid overcrowding. They note that the ‘minimally sufficient adjustment set would not change by including all possible associations with sex and age as source variables’. Figure reproduced with permission from Röhrig et al.^41^

Box 1. Directed acyclic graphs and the backdoor criterion help to identify confounders of the association between electrocardiographic findings and disability. In unadjusted and age- and sex-adjusted analyses, Röhrig *et al*.^41^ found an association between electrocardiographic (ECG) findings and disability status in older adults. They constructed the DAG in *Figure 2* and used an algorithm to identify a minimally sufficient adjustment set of covariates which satisfied the backdoor criterion. Their statistical adjustment set (age, sex, physical activity, obesity, diabetes mellitus, education, income, stroke, heart disease, and lung disease) blocks all confounding paths between ECG findings and disability status in *Figure 2* and does not block any causal paths between the two variables. When adjusting for these variables, they found that the association between ECG findings and disability status was completely explained away by confounding and concluded that ECG findings are not a cause of disability. Stepwise selection approaches applied to these data resulted in residual confounding and suggested an association between ECG findings and disability, thus demonstrating that data-driven approaches are inappropriate for causal modelling.

### Prediction research

Prediction research can be split into two categories concerning variable selection approaches. A researcher may be interested in predictor research, where the aim is to understand whether a single factor predicts an outcome (often referred to as the endpoint).^42–44^ Association estimates from such studies are reported as relative measures, e.g. a hazard ratio, risk ratio, or odds ratio.^44^ Such research can help identify groups of individuals with a shared characteristic that are at a higher risk of the outcome. Future research may then investigate strategies to prevent disease occurrence, improve/monitor prognosis, or screen for a disease within the high-risk group. Additionally, randomized trial design may be improved by ensuring treatment groups are balanced with respect to strong predictors of the trial outcome and adjusting for them.^42,45,46^ Further, such predictors may also be candidates for inclusion into multivariable prediction models.^47^

Alternatively, a researcher may be interested in prediction modelling, where the aim is to estimate an individual’s absolute risk/probability or value of the endpoint, often using a combination of predictors in a multivariable regression model.^17,47,48^ For example, QRISK3 (https://www.qrisk.org/) combines 21 predictors in a model that can estimate the risk of having a heart attack or stroke within the next 10 years for a UK patient without cardiovascular disease aged 25–84 years.^49^ The approaches to variable selection for predictor research and prediction model research are summarized below.

### Predictor research

Prediction research in medicine often involves predicting, or identifying predictors of, the occurrence of a disease or predicting the future health status of someone with a disease (i.e. prognosis).^22^ Predictors of the future health status in people with a disease are often referred to as prognostic factors, which may be useful for defining disease, monitoring disease progression, informing treatment recommendations, and more.^42^ Predictors of treatment effect (and other types of effect modifiers) are another type of predictor. They can be used to select the optimal treatment for people with a disease.^50^ Subgroups of patients determined by the existence of, or levels of, the predictor may receive different treatments according to how effective the treatment is in that population (i.e. stratified medicine).^50^ However, Hingorani *et al*.^50^ point out that not all prognostic factors are predictors of treatment effect and vice versa. All the types of predictors mentioned above can also be used as the building blocks for prediction models, which are described later in this article.

Riley *et al*.^42^ argue that predictor/prognostic factor research should be classed into three levels. Level 1 refers to exploratory studies which aim to identify new candidate predictors using data-driven methods. Such studies can help to generate hypotheses when little background knowledge is known about whether variables may be predictive or not; for example, when identifying new candidate biomolecular and genetic prognostic factors.^42^ Once candidate predictors are identified from Level 1 studies, there is a need for Level 2 hypothesis-confirming studies. Level 2 studies either can be used to confirm the findings from Level 1 studies or can be used to confirm hypotheses formed based on strong *a priori* biological reasoning. Once multiple independent Level 2 studies have been conducted, a Level 3 systematic review and meta-analysis can be conducted to synthesize the Level 2 evidence.

Due to the lack of background knowledge, variable selection in Level 1 studies is generally data-driven, often conducted using stepwise selection or univariable prefiltering. However, data-driven strategies applied to huge numbers of candidate predictors are more likely to result in chance associations due to multiple hypothesis testing, biased association estimates, exaggerated *P*-values, and underestimated measures of variation.^44^ Further, such data-driven approaches do not answer the desired clinical question of whether the variable adds predictive value over standardly used predictors. Instead, results from data-driven methods often have no clear clinical interpretation.

Additionally, it is far more clinically relevant to understand the magnitude of the association [along with the precision of the corresponding 95% confidence interval (CI)] instead of focusing on whether a variable is statistically significant (which is highly dependent on several clinically irrelevant factors, such as the study’s sample size).^51^ Due to the issues with Level 1 studies, they should be reported carefully, stating that their purpose is only to screen many candidate predictors to generate hypotheses that require confirmation in future independent research.^44^

The analytical approach to hypothesis-confirming studies should not be data-driven and should be based on existing knowledge, which can be identified from expert opinion or a literature review. Two questions may be of interest when investigating the predictive value of a variable in predictor studies. Firstly, one may be interested in whether the variable predicts the outcome by itself. This can be answered without the need for any statistical adjustment, i.e. in a univariable analysis.^52^ Secondly, a researcher may know that there are predictors that are either already known or already standardly used in practice.^52^ In this scenario, it would be helpful for the researcher to investigate the added predictive value of the new candidate predictor over and above the known, or standardly used, predictors. Note that, as mentioned in the introduction section, this is not the same as adjusting for confounders. The purpose of adjusting for confounders is to avoid biasing the causal effect estimate, whereas the purpose of adjusting for existing predictors is that the researcher wants to answer a different question, i.e. not only does the variable predict the outcome univariably, but does it predict the outcome over and above pre-existing predictors? One caveat to this is the scenario when the study seeks to determine whether a predictor should be intervened upon to reduce the risk of the endpoint. If the researcher is interested in whether or not to intervene on the predictor, then it is causal research, not prediction research, and causal inference methods (or, ideally, a randomized controlled trial design) should be used.

When conducting a Level 3 meta-analysis with traditional aggregated data methods, the reviewer is limited to synthesizing evidence from studies that may not have adjusted for standardly used predictors. Individual participant data meta-analyses have been suggested as an approach to overcome some shortcomings of the primary studies analyses since it allows for reanalysis, which can include statistical adjustment for existing predictors to determine the independent predictive value of the candidate predictor being investigated (if data on conventional predictors were collected in the component datasets).^53^

To investigate whether a variable is a predictor of treatment effect/effect modifier, appropriate methods (causal inference methods or the use of a randomized trial) are also required to reliably estimate the treatment effect. A variable is a predictor of treatment effect/effect modifier if the effect of the treatment/exposure varies according to strata of the predictor.^54^ The predictor does not need to have a causal effect on the outcome. Due to this fact, predictors of treatment effect/effect modifiers are referred to in this article as a type of predictor. This distinction also helps to distinguish the concept of effect modification from causal interaction, which relates to the effects of two interventions/exposures, which requires that the effects of both interventions/exposures are unconfounded.^54^ Thus, when assessing whether a variable is an effect modifier, no additional variables need to be adjusted for beyond those that are required to account for confounding of the exposure–outcome relationship. For more details about the distinction between causal interaction and effect modification, see VanderWeele.^54^

In summary, to establish whether a variable is a predictor of an outcome, no statistical adjustment is required. In circumstances where there are established, or routinely used, predictors, researchers should also present analyses adjusted for the established predictors to determine whether the candidate predictor provides independent predictive power (*Box 2*).

Box 2. Case study—C-reactive protein as a prognostic factor/biomarker in people with stable coronary disease. Hemingway *et al*.^55^ conducted a systematic review to evaluate whether C-reactive protein was an independent predictor (‘independent’ meaning over and above other known prognostic factors) of coronary or cardiovascular events, or all-cause mortality. From prior research and expert knowledge, they identified the following conventional prognostic factors: age, sex, smoking status, BMI, diabetes, lipid variables (total cholesterol, LDL, HDL, triglycerides), and inflammatory markers (fibrinogen, IL-6, white cell count). Three out of the 83 studies had adjusted for all conventional prognostic factors and yielded a relative risk of 1.5 (95% CI 1.3–1.9) when pooled in a meta-analysis. Thirteen studies had adjusted for all prognostic factors except inflammatory markers, with relative risk 1.7 (95% CI 1.4–2.0) and *I*^2^ 33.7% (95% CI 0.0–64.6). Across all 83 studies, an additional 78 variables that are not conventional prognostic factors were also adjusted for. When pooling all 83 studies, the relative risk was 2.0 (1.8–2.2), a 33% larger relative risk than when conventional prognostic factors were adjusted for; plus, an *I*^2^ of 79.5% (95% CI 75.1–82.8), a 136% increase in heterogeneity. The results of the review demonstrated that variable selection in predictor research is highly inconsistent and leads to heterogeneous results, which may exaggerate (or, in other scenarios, mask) the predictive ability of a variable. This demonstrates that *a priori* identification of, and subsequent statistical adjustment for, routinely used prognostic factors is necessary to improve analyses.^52,55^

### Prediction modelling

Using one predictor is generally inadequate to accurately predict a health-related outcome. Thus, a formal combination of predictors in a multivariable prediction model may be desirable. The aim of a prediction model is to estimate the absolute risk/probability or value of a specific endpoint for individual patients, satisfying pre-specified eligibility criteria.^47^ For example, in the QRISK3 calculator (*Box 3*), the model is intended for use only in UK patients aged 25–84 years without cardiovascular disease, and the endpoint, having a heart attack or stroke within the next 10 years, is specifically defined with respect to the diseases and the timepoint.^49^

Box 3. Case study—the QRISK2^79^and QRISK3 cardiovascular disease risk score calculators (https://www.qrisk.org/). QRISK2^79^ is a prediction model designed to estimate the 10-year risk of heart attack or stroke for UK patients aged 35–74, which was updated to become QRISK3^49^ in 2017 to include new predictors and expand inclusion criteria to 25–84-year-olds. Predictor variables were selected for inclusion into QRISK2 if there was existing evidence that the variable was a predictor of cardiovascular disease. No data-driven approaches were used to reduce the number of predictors. The predictors included are age, ethnicity, sex, smoking status, systolic blood pressure, cholesterol ratio, BMI, family history of coronary heart disease, deprivation, treated hypertension, rheumatoid arthritis, chronic renal disease, Type 2 diabetes, and atrial fibrillation.

QRISK3 included all variables from QRISK2 but also tested the inclusion of chronic kidney disease, systolic blood pressure variability, migraine diagnosis, corticosteroid use, systemic lupus erythematosus, second-generation atypical antipsychotic use, mental illness, HIV/AIDS, and erectile dysfunction. These variables were nominated due to new research or guidelines highlighting their potential predictive ability. Each of these candidate predictors was included if it had an adjusted hazard ratio of <0.90 or >1.10 (for binary variables) and was statistically significant at the 0.01 level. The strict 0.01 significance level and the requirement of a moderate to large association estimate were likely chosen to prevent unimportant variables from being selected due to the large sample size of 7.9 million people.

With technological advancements and the growth of ‘Big Data’, modern data sets can have a vast number of variables for a researcher to consider for inclusion. There are many strategies for selecting variables for prediction models, which are described in detail in statistics textbooks,^16,17^ but there is no consensus on which approach is best. This section provides an overview with recommendations and describes common mistakes in model building.

Compared with causal modelling, the approach to prediction modelling is often much more data driven. However, some key considerations must be made before data-driven methods are considered. The first step to identifying candidate predictors should be to conduct a literature review and seek expert advice about which variables are already known to be predictors of the outcome.^16,17^ Data-driven approaches have many limitations, which are described below, so pre-specifying predictors that have already been proven to be predictive can help avoid many of these issues.^16^

If some candidate predictors are highly correlated, they should not all be included in the model since this could result in multicollinearity and overfitting. For example, BMI, height, and weight could be reduced to only BMI. Principle component analysis may help identify the essential features of high-dimensional data, thus reducing the number of variables without greatly reducing the ability to explain data variation.^56^

Additionally, clinical utility should also be considered when nominating candidate predictors. For a model to be easy to use in practice, variables should be clearly defined, the assessment of the variable should be standardized, and the variable should be easy to measure.^57^

One approach to model building would be to include all candidate variables that were identified from the background knowledge criteria above, and make no further reduction in the number of variables.^16^ This approach has been suggested to reduce overfitting, selection bias, and the biasing of standard errors and *P*-values.^16^ However, in some circumstances, there may be uncertainty surrounding the background knowledge of which variables are predictors, or the number of candidate predictors may be so large that the use of the prediction model would be impractical. In such circumstances, data-driven methods may be opted for to further reduce the number of variables.^16^

Univariable selection, i.e. estimating univariable models for each candidate predictor and including all variables that are statistically significant at some pre-defined threshold, is a simple approach to selecting models. This approach can lead to rejecting important predictors that only become significant after the inclusion of other predictors,^58,59^ as well as failing to identify the predictive power of a variable over and above other included predictors.

Stepwise selection criteria (an automated iterative algorithm involving adding or removing variables based on whether they are statistically significant) are easily implemented and commonly used. However, stepwise selection has been heavily criticized and leads to the apparent performance of the selected model being overestimated.^16,17,60,61^

Both univariable and stepwise approaches share many of the same problems related to their reliance on statistical significance, which is prone to a number of issues.^51,62^ Selection based on statistical significance leads to overestimation of coefficients since predictors are more likely to be selected if their coefficients are large; although, they may be larger than they should be due to chance, especially in small datasets.^61^ When a large number of candidate predictors are being tested for inclusion, there are also issues with multiple hypothesis testing leading to the false inclusion of variables that provide limited additional predictive abilities.^63^ In large datasets, variables with statistically significant, but very small, association estimates may be included in the model, leading to a non-parsimonious model, which some researchers attempt to overcome by excluding variables with association estimates under a certain magnitude. Variables may also be incorrectly excluded by univariable and stepwise approaches due to insufficient statistical power in small datasets, especially if strict statistical significance thresholds are used.^60,64,65^

Best subset criteria are an alternative to methods based on statistical significance. If there are *p* candidate predictors, then 2*^p^* models are fit using each possible combination of the candidate predictors. Then, the best model is selected based on some information criterion, most commonly the Akaike information criterion or Bayesian information criterion.^66,67^ Best subset criteria are more well-suited to prediction modelling than statistical significance criteria since they focus on the predictive power of a collective set of variables, as is its intended use, rather than individually assessing each predictor for inclusion/exclusion.

When data-driven methods are used to derive a model, especially when the number of candidate predictors is large or the sample size is small, there is a risk that model coefficients are inflated. It is recommended to adjust for this using a shrinkage technique; otherwise, model predictions may be too extreme.^16^ Additionally, shrinkage is recommended to reduce overfitting in model development to improve performance in new data.^68^ One common approach is to apply a post hoc uniform shrinkage factor (which can be estimated by bootstrapping) to all predictor coefficients produced using one of the variable selection methods mentioned above. Another common option is to use a penalized regression method,^69^ such as Least Angle Selection and Shrinkage Operator, which shrinks coefficients and conducts variable selection all in one process. There is no clear evidence of one approach being superior,^70^ but it is recommended to perform some form of shrinkage to account for overfitting.^71^

After a prediction model has been developed, it is critical to assess the model’s performance, i.e. how accurate the model’s predictions are. Internal validation^72^ assesses the model’s performance in data from the same source from which it was developed, e.g. using bootstrapping or cross-validation. External validation^73^ tests the model’s performance in data from a new source, e.g. data from a different hospital or a different electronic health database. A model’s performance is usually assessed based on measures of calibration (i.e. how well the model’s predicted values/probabilities agree with observed values/probabilities) and discrimination (i.e. how well the model’s predictions differ between those with different outcome values).^74^ Testing of model performance is critical in prediction model research; however, it is often not possible to conduct in causal modelling, where the aim is to estimate a causal effect, since the true causal effect is unknown.

When conducting predictor research, the aim of the research is to determine whether the variable is a predictor of a future outcome by estimating its relative association with the outcome, not to accurately estimate an individual’s absolute risk/probability or value of the endpoint. Predictor research can be helpful to identify high-risk groups; however, often, a single predictor will not be enough to accurately predict an individual’s endpoint. If the aim is to use a single predictor to accurately estimate the risk or value of the endpoint, then the researcher is using the model as a prediction model. As with any prediction model, validation is required to test the model’s performance.

A common misunderstanding in the interpretation of prediction model results is that researchers may mistakenly believe that they can interpret a coefficient from a prediction model causally.^4^ That is, they may calculate the absolute risk of the outcome before and after changing the inputted value of a predictor and believe that this would equate to how much the individual could reduce their risk if they were to change their lifestyle circumstances in this way. However, prediction models are not designed with the intention to eliminate confounding, avoid collider bias, or keep causal paths open. Thus, the other variables that are included in the model will likely not satisfy the backdoor criterion with respect to the predictor and outcome in question, and so the coefficient should not be interpreted causally. Counterfactual prediction modelling (predicting outcomes in hypothetical scenarios in which the values of predictors can be changed through intervention) is a developing area, and it can be read about more in Dickerman *et al*.,^75^ Prosperi *et al*.,^76^ and van Geloven *et al*.^77^ It should also be noted that, whilst not all predictors of an outcome will be causes of that outcome, all causes of an outcome will be predictors of that outcome (even if they are not strongly predictive). It has been shown that selecting causes of an outcome for inclusion in a prediction model may help improve model transportability.^78^

### Descriptive epidemiology

Variable selection in descriptive epidemiology may be described as a case of ‘less is more’; often with there being little need to incorporate many additional variables into analyses. In their framework for descriptive epidemiology, Lesko *et al.*^80^ argue that there are two main reasons why a researcher may want to account for additional variables in descriptive research. Firstly, one may want to stratify analysis to characterize the outcome distribution with respect to an additional variable. For example, during the COVID-19 pandemic, information about how infection rates differed according to geographical regions helped guide policy decisions. COVID-19 death rates were also described according to personal characteristics such as age, gender, ethnicity, comorbidities, socioeconomic status, and more.^81–83^ Such information helped policymakers to know where to place limited resources (e.g. vaccines) for maximum impact and also helped motivate future research; for example, researching the reasons for increased death rates in these populations (i.e. causal research) or helping to build prognostic models to predict the outcomes for people with COVID-19 (i.e. prediction research). A second reason that a researcher may want to account for an additional variable in descriptive research is to account for some nuisance variable over which the study sample differs from the target population, e.g. age standardization may be conducted so that the age structure of the study sample is the same as the target population.^84^ Whilst age standardization is a common approach in descriptive epidemiology for comparing health outcomes across populations with different age structures, it should be noted that this approach does not describe actual rates.^85^ Instead, the approach produces relative indices that should be used for comparison only.^85^ The comparison should also not be interpreted as being what would be observed in a counterfactual world in which the age structure is different, as causal assumptions (e.g. no confounding) need to be satisfied to draw such a conclusion about the counterfactual standardized population.^14^

The authors have highlighted that descriptive studies often suffer from overadjustment, due to researchers believing that they must account for confounding variables.^2,12^ Statistical adjustment for confounding variables aims to avoid bias when estimating a causal effect. However, estimating a causal effect is not the aim of a descriptive study; rather, the descriptive study aims to describe the distribution of a health outcome in a well-defined target population. Accounting for some additional variable in a descriptive study serves the purpose of describing the distribution of the health outcome in more specific subgroups, in the case of stratified analyses, or to account for a nuisance variable so that the study sample more closely represents the target population.^80^ Thus, the choice to account for a stratification variable or a nuisance variable should be guided by an understanding of which population the researcher is attempting to describe, what question the researcher is attempting to answer, and what the purpose of the study is (*Table 1*).^2^

**Table 1:** Summary of variable selection approaches for different types of medical research

Type of research	Aim of research	Real-world importance	Variable selection approach	Reason to account for variables	Consequences of incorrect method
Causal/explanatory	To understand the causal mechanism that leads to a health-related outcome. To estimate the causal effect of an exposure on an outcome.	To identify a target for intervention or assess the effectiveness of an intervention. To understand aetiology.	Identification of confounders through background knowledge. DAGs and the backdoor criterion may be used to depict assumptions and determine if the statistical adjustment set is sufficient.	To block non-causal pathways, which in turn prevents the estimation of non-causal associations.	The causal effect estimate may be biased, which may lead to incorrect conclusions about whether a variable does or does not cause the outcome or false information about the magnitude of the effect.
Predictor	Identify a predictor of the occurrence of a disease, the future health status of someone with a disease (i.e. a prognostic factor), or the likely response to a treatment (i.e. a predictor of treatment effect).	Predict the occurrence of disease: motivating future causal studies or identifying candidate predictors in prediction models. Prognostic factors: defining disease, monitoring disease progress, informing treatment recommendations, candidate predictors in prediction models, potential targets for intervention to improve prognosis, aiding the design of RCTs. Predictors of treatment effect: stratified medicine.	Unadjusted analysis, or statistical adjustment for existing, or standardly used, predictors identified through expert opinion and literature review.	To determine the added predictive value of the candidate predictor over and above known predictors.	Results may be difficult to interpret clinically, or incorrect conclusions may be made about the variable's predictive power.
Prediction model	Predict the occurrence of a disease, the future health status of someone with a disease (aka a prognostic model), or another health-related outcome.	Accurately predict the value or risk of a health-related outcome to identify high-risk individuals to inform clinical decision-making.	A combination of: Identifying known predictors from literature review and expert opinion. Considering clinical usability. Data-driven variable selection techniques. Shrinkage of coefficients.	Variables are not ‘accounted for’ or ‘adjusted for’. The set of variables is chosen based on whether they *collectively* are best at predicting the endpoint.	Poor predictive performance (which should be tested through internal and external validation.) Poor clinical utility.
Descriptive	Quantitatively describe the distribution of disease (or other features of health) in a population.	Analyse trends, generate hypotheses for analytical studies, or guide policymakers in planning health services and deciding how to utilize resources.	Stratification variables are selected based on which research question the investigator seeks to answer. Nuisance variables may be selected based on how the study sample differs from the target population.	Stratified analysis seeks to characterize the outcome distribution with respect to an additional variable. Standardization aims to account for a nuisance variable for which the study sample differs from the target population.	Difficulty in interpreting results.

## Discussion

There is a growing appreciation that different types of research questions require different methodological approaches. This paper summarizes the key differences in variable selection methods between causal modelling, predictor/prognostic factor research, prediction modelling, and descriptive epidemiology. Causal models require statistical adjustment for confounders to avoid confounding bias, whereas the purpose of accounting for additional variables in prediction modelling or descriptive research is not to avoid confounding bias, as is commonly misunderstood in scientific literature. In predictor research, additional variables may be adjusted for to examine the added predictive value of the candidate predictor over and above existing predictors. In prediction modelling, there is not one main predictor that is the focus of the research; rather, prediction models are a formal combination of many predictors that work collectively to estimate the absolute risk of the endpoint. Descriptive studies may choose to standardize over a nuisance variable so that the study sample has the same distribution, with respect to this variable, as the target population. Additionally, pre-specified stratification variables may be selected to further characterize the outcome distribution in descriptive research.

This paper highlights that the teaching of statistical and epidemiological methods should be conducted in a unified framework to encourage an appreciation that different methods are required for causal inference, predictor research, developing prediction models, and descriptive epidemiology.

## Data Availability

No new data were generated or analysed in support of this research.

## Lead author biography

Dr Brett Dyer is a research fellow in Biostatistics at Griffith University (Australia). In 2018, he won a Royal Statistical Society (RSS) prize for being the top-performing student on an RSS-accredited master’s degree when studying at the University of Leeds (UK). In 2022, he completed his PhD at Keele University (UK) and has since worked as an academic consulting biostatistician at Griffith University. His methodological research expertise centres on developing predictive and causal models for observational data.
